# Identifying risk factors of developing type 2 diabetes from an adult population with initial prediabetes using a Bayesian network

**DOI:** 10.3389/fpubh.2022.1035025

**Published:** 2023-01-12

**Authors:** Pilar Fuster-Parra, Aina M. Yañez, Arturo López-González, A. Aguiló, Miquel Bennasar-Veny

**Affiliations:** ^1^Department of Mathematics and Computer Sciences, Balearic Islands University, Palma, Spain; ^2^Institut d'Investigació Sanitària Illes Balears (IdISBa), Hospital Universitari Son Espases, Palma, Spain; ^3^Department of Nursing and Physiotherapy, Balearic Islands University, Palma, Spain; ^4^Research Group on Global Health and Human Development, Balearic Islands University, Palma, Spain; ^5^Escuela Universitaria ADEMA, Palma, Spain; ^6^Prevention of Occupational Risk in Health Services, Balearic Islands Health Service, Palma, Spain; ^7^CIBER de Epidemiología y Salud Pública (CIBERESP), Instituto de Salud Carlos III (ISCIII), Madrid, Spain

**Keywords:** Bayesian networks, Markov blanket, prediabetes, type 2 diabetes, prevention, risk factors

## Abstract

**Background:**

It is known that people with prediabetes increase their risk of developing type 2 diabetes (T2D), which constitutes a global public health concern, and it is associated with other diseases such as cardiovascular disease.

**Methods:**

This study aimed to determine those factors with high influence in the development of T2D once prediabetes has been diagnosed, through a Bayesian network (BN), which can help to prevent T2D. Furthermore, the set of features with the strongest influences on T2D can be determined through the *Markov blanket*. A BN model for T2D was built from a dataset composed of 12 relevant features of the T2D domain, determining the dependencies and conditional independencies from empirical data in a multivariate context. The structure and parameters were learned with the bnlearn package in R language introducing *prior* knowledge. The *Markov blanket* was considered to find those features (variables) which increase the risk of T2D.

**Results:**

The BN model established the different relationships among features (variables). Through inference, a high estimated probability value of T2D was obtained when the body mass index (BMI) was instantiated to *obesity* value, the glycosylated hemoglobin (HbA1c) to more than 6 value, the fatty liver index (FLI) to more than 60 value, physical activity (PA) to *no* state, and age to 48–62 state. The features increasing T2D in specific states (warning factors) were ranked.

**Conclusion:**

The feasibility of BNs in epidemiological studies is shown, in particular, when data from T2D risk factors are considered. BNs allow us to order the features which influence the most the development of T2D. The proposed BN model might be used as a general tool for prevention, that is, to improve the prognosis.

## Introduction

Type 2 diabetes (T2D) is recognized as a global serious health concern with a considerable impact on human life and health expenditures (1) whose prevalence has steadily increased and is now one of the main causes of morbidity and mortality in adults (2, 3). T2D is a preventable condition and is very likely to develop in people whose blood glucose levels are higher than normal but do not fulfill the criteria for a diagnosis of T2D (prediabetes) (4). Prediabetes also increases the risk of cardiovascular disease and mortality (5), similar to people with diabetes (6). The rate of progression to T2D for this population ranges between 5 and 10% annually (7, 8). According to the IDF Diabetes Atlas, a total of 6.0% of the worldwide population (463 million individuals aged 20–79 years) are estimated to have prediabetes (9). Acting on prediabetes could be a window of opportunity to prevent or delay T2D. Different risk factors for the progression from prediabetes to T2D have been described such as obesity (general or abdominal), a family history of diabetes, ethnicity, gestational diabetes, high systolic blood pressure (SBP), low level of high-density lipoprotein cholesterol (HDL-C), and tobacco smoking (4, 10, 11). More studies and data analysis frameworks are needed to evaluate the complex relationship among different risk factors on the progression to T2D. In this sense, a model for T2D based on Bayesian networks (BNs) is considered.

Models, such as BNs (12–14), capture the potential relationships among features (factors) like an expert understands them (15) by a directed acyclic graph (DAG) where the nodes have conditional probability tables. They constitute an established framework and an efficient reasoning tool for uncertainty management in artificial intelligence (AI), which has been used to discover the relationships between variables determining the direct and indirect dependencies (14, 16, 17). Models include graph theory together with probability theory in order to represent the relationships between variables (18), and they are especially selected because they provide probability estimates rather than predictions. Moreover, BNs offer a paradigm for interpretable AI, where high-stakes applications have increased, and therefore, the use of interpretable models is important (19). In this sense, they can be applied to help health practitioners by providing T2D characterization estimates as a probability network that can be continuously updated according to patient information given by practitioners.

A BN (12, 13) B encodes a joint probability distribution *P* over a vector of random variables (features) **X** = (*X*_1_, …, *X*_*n*_), and it consists of (20) (i) a DAG G, which is composed of a set of variables (features), each variable has a finite set of mutually exclusive states, where these variables are the vertices (i.e., nodes) of G, and a set of directed edges (i.e., arcs) between these variables (features) of vector **X**, and (ii) a set of parameters θ such that B=(G,θ), where G denotes a structure represented by a DAG, θ is a set of local parameters according to the structure G, the parameters are the conditional probability distributions for the values of each variable given different value combinations of their parent nodes. The joint probability distribution *P* encoded by the BN B factorizes as a product of several local conditional distributions which denotes the dependency/independency structure by a DAG:


(1) $$\begin{array}{l} P\left(X_{1},\ldots ,X_{n}\right)=\overset{n}{\underset{i=1}{\prod }}P\left(X_{i}|pa\left(X_{i}^{G}\right)\right). \end{array}$$

Equation (1) is also referred to as the *chain rule for BNs*, where $pa\left(X_{i}^{G}\right)$ denotes the parent nodes of *X*_*i*_ in DAG G, which is the main reason for the formulation of a multivariate distribution by BNs.

**Figure 1:**
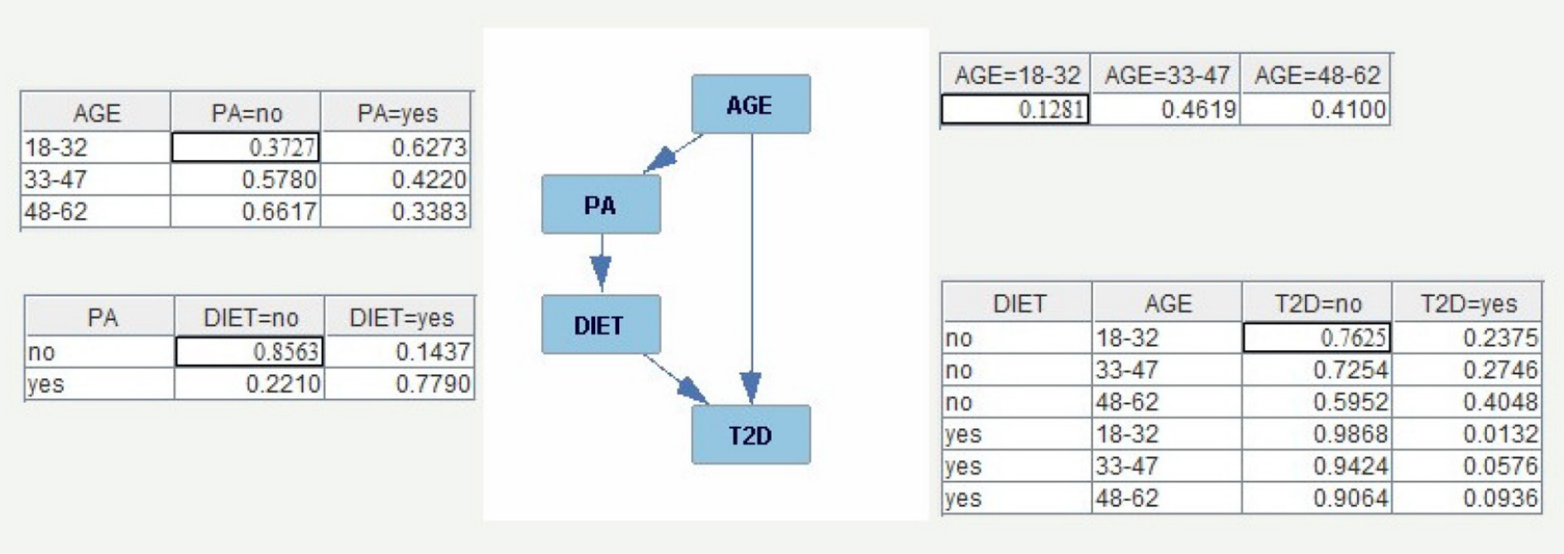
Example of BN with the conditional probability tables of each node. In the Figure P(AGE = 18–32) = 0.1281, P(PA = no∣AGE = 18–32) = 0.3727, P(DIET = no∣PA=no) = 0.8563, and P(T2D = no∣AGE = 18–32, DIET=no) = 0.7625.

An example of BN, including the structure and conditional probabilities tables, is presented in Figure 1. In this case, the chain rule is given by


$$\begin{array}{l} P\left(AGE,PA,DIET,T2D\right)=P\left(AGE\right)\cdot P\left(PA|AGE\right)\\ \;\cdot P\left(DIET|PA\right)\cdot P\left(T2D|DIET,AGE\right). \end{array}$$

Bayesian networks are used to make an inference (21); therefore, the basic concepts for inference flow when new information is introduced in a BN are presented here.

**Figure 2:**
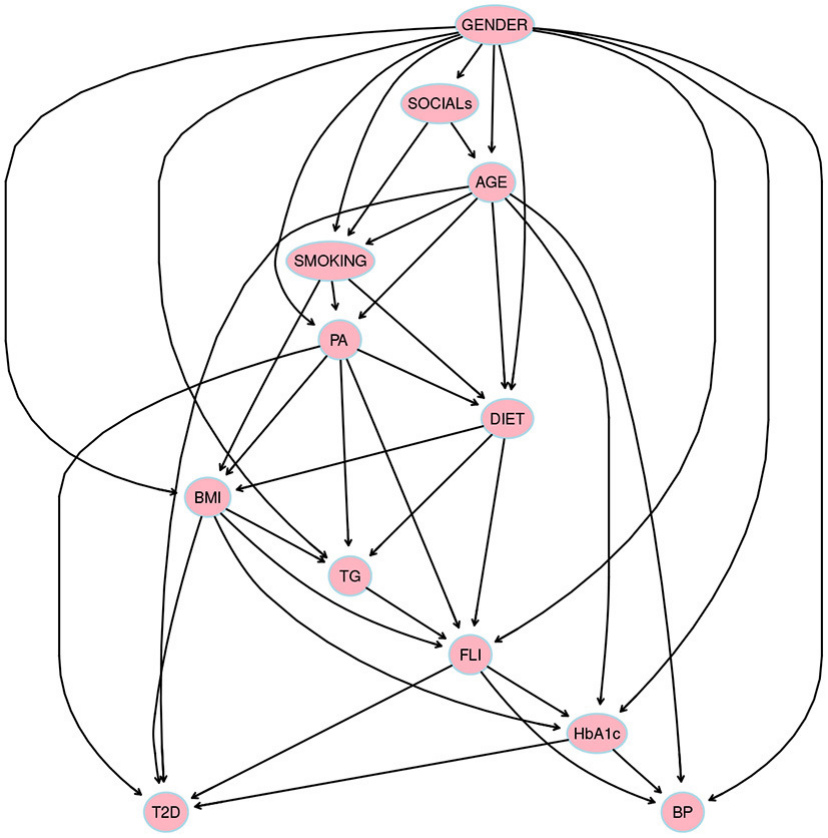
The structure obtained through hill climbing learning algorithm *hc* from *bnlearn* package in R language using a threshold = 0.85 by *model averaging* over 500 networks. Prior knowledge was included in model selection, thus variables were divided into four blocks: 1) *background variables* = {*GENDER, AGE*, and *SOCIALs*}, 2) *conditional variables* = {*DIET, SMOKING, PA*, and *BMI*}, 3) *intermediate variables* = {*HbA1c, FLI, BP*, and *TG*}, and, 4) *diagnostic variable* = {*T2D*}.

Two variables *X* and *Y* in a BN are *d-separated* if, for every possible path between *X* and *Y*, there is an intermediate variable *Z* such that either (i) the connection is serial (*X* → *Z* → *Y* or *X* ← *Z* ← *Y*) or diverging (*X* ← *Z* → *Y*) and *Z* is instantiated, or (ii) the connection is converging (*X* → *Z* ← *Y*) and neither *Z* nor any of *Z*'s descendants have received evidence. It seems necessary to know when influence flows from a node *X* to another node *Y via* a node *Z*, when this occurs, it is said that the trail *X ⇌ Z ⇌ Y* is active. A causal trail *X* → *Z* → *Y* (serial connection), an evidential trail *X* ← *Z* ← *Y* (serial connection) or, a common cause trail *X* ← *Z* → *Y* (diverging connection) is active if and only if Z is not observed. A common effect trail *X* → *Z* ← *Y* (converging connection) is active if and only if either *Z* or one of *Z*'s descendants is observed. For instance, in Figure 2, there are two possible paths between *PA* and *T*2*D*, i.e., *PA* → *DIET* → *T2D* and *PA* ← *AGE* → *T2D*, when *DIET* and *AGE* are instantiated then *PA* and *T*2*D* would be *d-separated*.

The Bayesian network B=(G,θ) satisfies the local Markov condition if, for each variable (feature) *X*_*i*_, *X*_*i*_ is conditionally independent of the set of all its non-descendants given the set of all its parents. The global Markov property states that any node *X*_*i*_ is conditionally independent of any other node given its *Markov blanket*, i.e., *I*(*X*_*i*_, *non-markov-blanket*(*X*_*i*_)∣*markov-blanket*(*X*_*i*_)); the *Markov blanket* of a node includes its parents, its children, and the children's other parents (spouses). Any node in the BN B would be *d-separated* from the nodes belonging to the non-Markov blanket given its Markov blanket.

The Bayesian network models have been widely used successfully in different fields, such as diagnostic diseases (14, 22–28), neuroscience (15, 29), analysis of complex disease systems (30–32), clinical decision support (33, 34), human immunodeficiency virus (HIV) and influenza research (35, 36), and even in interactions between multiple diseases (37).

## Methods

###  Participants

The present study examined a cohort of Spanish working adults (*n* = 16, 648, Men: 12,080, Women: 4,568), who had prediabetes at baseline. Participants were selected from the working population of 234, 995 potentially eligible individuals, belonging to different productive sectors (public administration, construction, healthcare, service industries, or postal services) who received occupational health examinations between 2012 and 2013. Criteria for selecting the subjects were as follows: age between 20 and 65 years and FPG between 100 and 125 mg/dl, according to ADA criteria (38). Exclusion criteria were FPG ≥ 126 mg/dl or HbA1c ≥ 6.5% at baseline, current treatment with oral antidiabetic or diagnosed with T2D, anemia, current treatment with systematic glucocorticoid, cancer treatment, and pregnancy. All subjects underwent a standard health examination, sociodemographic characteristics, anthropometric measurements, lifestyle, and clinical data at baseline. Follow-up examinations were performed at 5 years (2017–2018). The study protocol was conducted in accordance with the Declaration of Helsinki with human participants and was approved by the Institutional Review Board of the Balearic Islands Health Research Ethics Committee (CEI-IB Ref. No: 1887). All participants were informed of the purpose of the study before they gave consent to participate.

###  Data collection and definition of variables

Most methods have been described in greater detail previously (39, 40). The following data were collected: sociodemographic variables, including age, gender, education level, and social status. Social status was ascertained using the Spanish adaptation of the Goldthorpe classification suggested by the Spanish Epidemiology Society classification (41).

Anthropometric measurements: All anthropometric measurements were performed by well-trained technicians and were made according to the recommendations of the International Standards for Anthropometric Assessment (ISAK) (42). Body weight was measured to the nearest 0.1 kg using an electronic scale (Seca 700 scale, Seca gmbh, Hamburg). Height was measured to the nearest 0.5 cm using a stadiometer (Seca 220 Telescopic Height Rod for Column Scales, Seca gmbh, Hamburg). BMI was calculated as weight (kg) divided by height (m) squared (kg/*m*^2^). Participants were categorized depending on their BMI following WHO criteria: normal weight (BMI = 18.5–24.9 kg/*m*^2^), overweight (BMI = 25.0–29.9 kg/*m*^2^), and obese (BMI ≥ 30.0 kg/*m*^2^) (43). Waist circumference (WC) was measured half-way between the lower costal border and the iliac crest, using a flexible steel tape (Lufkin Executive Thinline W 606). Systolic (SPB) and diastolic blood pressure (DBP) were measured in triplicate, with a 1 min gap between measurements, using an electric and calibrated sphygmomanometer (OMRON M3, Healthcare Europe, Spain). Hypertension was defined as SBP ≥ 140 mmHg, or DBP ≥ 90 mmHg, or taking antihypertensive medication.

Blood samples were taken at baseline and 5 years. Venous blood samples were collected from the antecubital vein after a 12 h overnight fast in suitable vacutainers without anticoagulants to obtain serum. Serum concentrations of glucose, TG, GGT, and cholesterol were measured by standard procedures using an autoanalyzer (SYNCHRON CXⓇ9 PRO, Beckman Coulter clinical system, Brea, CA, USA). High triglycerides (TG) was defined as ≥ 150 mg/dl, and high cholesterol was defined as ≥ 200 mg/dl.

Fatty liver index (FLI) was used as a surrogate measure of fatty liver. FLI is a validated risk score system based on routine measurements of TG and gamma-glutamyl transferase (GGT) concentrations, WC, and BMI (44), and accurately identifies non-alcoholic fatty liver disease (NAFLD) and hepatic steatosis in the general population (40, 45).

Participants were asked if they engaged in moderate and/or vigorous physical activity (PA) at least 150 min/week according to WHO recommendations and if they consumed vegetables and fruits daily. Participants, also, were classified as non-smokers, current smokers, or former smokers, according to WHO criteria.

Prediabetes was defined as FPG between 100 and 125 mg/dl according to the ADA criteria (38). Incident T2D was defined as the clinical diagnosis of T2D, FPG ≥ 126 mg/dl, or the initiation of anti-hyperglycemic treatment at follow-up.

###  Bayesian networks

In the process of learning a BN, the following steps have to be carried out: i) *structural learning* that will determine the topology of the BN or DAG and ii) *parametric learning* or estimation of conditional probabilities among the nodes once the network topology is established.

###  Structural learning

The problem of discovering the causal structure increases with the number of variables (46–48). Table 1 shows a description of the 12 variables considered.

**Table 1:** Description of 12 data set features used to learn the structure.

**Variable name**	**Description**	**Values**
*GENDER*	Male and Female	Men, Women
*T2D*	Type 2 diabetes	Yes, No
*SOCIALs*	Social status	I, II, III
*SMOKING*	Never, Former or current smoker	No, Former smoker, Yes
*PA*	Physical activity (At least 150 min/week)	Yes, No
*DIET*	Daily consumption of fruits and vegetables	Yes, No
*HbA1c*	Glycosylated haemoglobin	Less 6.0, More 6.0
*FLI*	Fatty liver index	Less 30, 30–60, More 60
*BMI*	Body mass index (kg/m^2^)	Underweight, Normal weight,
		Overweight, Obesity
*BP*	Blood pressure (mmHg)	Normal, High, Grade 1, Grade 2
*TG*	Triglycerides (mg/dl)	Normal, Limit, Hyper
*AGE*	Age interval in years	18-32, 33-47, 48-62

Basically, two approaches to structural learning can be considered (49): (i) *search-and-score* structure learning and (ii) *constraint-based* structure learning. Search algorithms based on search and score assign a number (score) to each BN, and then the BN structure with the highest score is selected. Constraint-based search algorithms determine a set of conditional independence analyses on the data (50), which is used to generate an undirected graph, then additional independence tests have to be considered in order to obtain the BN structure.

The package *bnlearn* (51, 52) of R language (53) was used to learn the structure, where prior knowledge of the variables under study was taken into account in order to reduce the number of structures that are consistent with the same set of independencies and to choose a structure which reflects the causal order and dependencies. The following blocks of variables were considered: i) *background variables* = {*Gender, Age*, and *Social status*}, ii) *conditional variables* = {*Diet, Smoking, PA*, and *BMI*}, iii) *intermediate variables* = {*HbA1c, FLI, BP*, and *TG*}, and iv) *diagnostic variable* = {*T2D*}.

By blacklisting arrows that point from a later block to an earlier block, the process of model selection was restricted (54). There are two possible options to obtain the structure: either select a *single best* model or else obtain some average model, which is known as *model averaging* (55). The model (see Figure 2) was learned with *hill-climbing (hc)* algorithm, a score-based algorithm that explores the search space starting from a network structure (usually the empty graph) and adding, deleting, or reversing one arc at a time until the score can no longer be improved (this process is also known as greedy search). The score used by the structure learning algorithm was the Akaike Information Criterion (AIC).

###  Parametric learning

Given the topology of the network, parameters were also obtained with the *bnlearn* package in R language by performing a Bayesian parameter estimation using the Dirichlet distribution (56).

A conditional probability distribution is obtained for each node. An example of conditional probability distribution is shown in Table 2.

**Table 2:** Expected values of probabilities for *age* feature conditional on combinations of its parent values, in this case, conditional on *gender*, and *social status* features.

**Gender**	**Social status**	**Age = *18–32***	**Age = *33–47***	**Age = *48–62***
Men	I	0.0681	0.4301	0.5018
Men	II	0.0726	0.4716	0.4558
Men	III	0.1430	0.4682	0.3887
Women	I	0.1147	0.5301	0.3552
Women	II	0.1163	0.5143	0.3694
Women	III	0.1306	0.4279	0.4416

###  T2D model

This T2D model allow us to obtain conditional independencies among the variables. In a BN, any node is conditionally independent of its non-descendants given its parents' nodes, i.e., *I*(*X, non*−*descendants*(*X*)∣*Pa*(*Xi*)), as the local Markov property states. For instance, in the BN obtained for T2D model which structure is given in Figure 2, we show some independencies:


$$\begin{array}{l} I(FLI,\{SMOKING,AGE,SOCIALs\}|Pa(FLI)\\ \;=GENDER,DIET,PA,TG,BMI),\\ I(HbA1c,\{TG,DIET,PA,SMOKING,SOCIALs\}|Pa(HbA1c)\\ \;=GENDER,AGE,FLI,BMI), \end{array}$$

$$\begin{array}{l} I(TG,\{SMOKING,AGE,SOCIALs\}|Pa(TG)\\ \;=DIET,PA,GENDER,BMI),\\ I(BP,\{T2D,TG,BMI,DIET,PA,SMOKING,SOCIALs\}\text{​​}|\text{​​}Pa(BP)\\ \;=GENDER,AGE,HbA1c,FLI),\\ I(T2D,\{BP,TG,DIET,SMOKING,SOCIALs,GENDER\}\\ \;|Pa(T2D)=PA,AGE,BMI,FLI,HbA1c). \end{array}$$

The *Markov blanket* of the diagnostic feature T2D is composed of five features *AGE, BMI, FLI, HbA1c*, and *PA*. According to the Global Markov property, T2D will remain independent of any other node (in the DAG) given the features that compose its Markov blanked, i.e., once the features in the Markov blanket are instantiated. The Markov blanket of a specific node (in a BN) determines the only features that influence such a node. However, the influence of features such as habit smoking (SMOKING), physical activity (PA), or dietary habits (DIET) may be of interest. In the flow of influence between SMOKING and T2D, the following causal trails in the DAG may be of interest: i) *SMOKING* → *BMI* → *T*2*D*; ii) *SMOKING* → *BMI* → *FLI* → *T2D*; iii) *SMOKING* → *PA* → *BMI* → *T*2*D*; iv) *SMOKING* → *PA* → *DIET* → *TG* → *FLI* → *T*2*D*; v) *SMOKING* → *BMI* → *HbA1c* → *T2D*; or vi) *SMOKING* → *PA* → *DIET* → *FLI* → *HbA1c* → *T*2*D*. The obtained model allow us to rank the features from the Markov blanket of T2D led to maximization of the probability of the T2D variable in the Yes state (see Table 3).

**Table 3:** Ranking the features from the Markov blanket of *T2D* led to maximization of the probability of the T2D variable in the *Yes* state, where in the initial BN without introduction of evidence *T2D* in *Yes* state reached a probability (expressed in percentage) of 22:3%.

**Rank**	**Instantiated variable**		**Value**	**T2D = Yes**
1	*BMI*	=	Obesity	67.3%
2	*HbA1c*	=	More than 6.0	63.5%
3	*FLI*	=	More than 60	56.4%
4	*PA*	=	No	37.2%
5	*AGE*	=	48–62	30.7%

###  Validation of the BN

The Bayesian network was validated using a 10-fold cross-validation for BN, using a log-likelihood loss function, obtaining an expected loss of 8.0470. In Table 4, the area under the ROC curve (AUC) and the percentage correctly classified for the different features is shown.

**Table 4:** AUCs and percentage correctly classified for the different features.

**Variable name**	**State**	**AUC**	**Accuracy**
GENDER	Men	0.8100	77.62
GENDER	Women	0.8099	77.62
T2D	Yes	0.9829	94.56
T2D	No	0.9825	94.56
SOCIALs	I	0.5973	78.86
SOCIALs	II	0.5928	78.86
SOCIALs	III	0.5952	78.86
SMOKING	Former smoker	0.6552	48.82
SMOKING	Yes	0.6229	48.82
SMOKING	No	0.6165	48.82
PA	No practice	0.9685	92.24
PA	Practice	0.9685	90.24
DIET	Yes	0.9037	83.52
DIET	No	0.9039	83.52
HbA1c	Lower 6.0	0.8415	86.49
HbA1c	Upper 6.0	0.8419	86.49
FLI	Lower 30	0.9596	80.45
FLI	30-60	0.8655	80.45
FLI	Upper 60	0.9683	80.45
BMI	Underweight	0.9042	78.23
BMI	Normal weight	0.9381	78.23
BMI	Overweight	0.8476	78.23
BMI	Obesity	0.9588	78.23
BP	Normal	0.7137	49.60
BP	High	0.5742	49.60
BP	Grade 1	0.6723	49.60
BP	Grade 2	0.7367	49.60
AGE	18–32	0.7443	55.72
AGE	33–47	0.6281	55.72
AGE	48–62	0.7076	55.72
TG	Normal	0.8949	76.47
TG	Limit	0.7806	76.47
TG	Hyper	0.9031	76.47

###  Performance comparison

Other classification performances (see Table 5) have been included in order to have reference benchmarks with respect to our BN, in particular, we include naïve bayes (NB), random forest, multilayer perceptron (MLP), and the ID3 algorithms WEKA (57). The performance of each classification model is evaluated using four statistical measures: accuracy, sensitivity, specificity, and ROC area.

**Table 5:** Performance for T2D feature comparing our BN and using a 10-fold cross validation experiments with the corresponding algorithms.

**Algorithms**	**Accuracy**	**Sensitivity**	**Specificity**	**ROC area**
Bayesian network	94.5639	0.9455	0.8268	0.9826
Logistic regression	94.3589	0.9440	0.83429	0.9740
Naïve Bayes	90.7196	0.9070	0.9160	0.9740
Random forest	94.2636	0.9430	0.8770	0.9670
Multilayer perceptron	94.4918	0.9450	0.8870	0.9440
ID3	93.6389	0.9440	0.8720	0.9170

## Results

**Figure 3:**
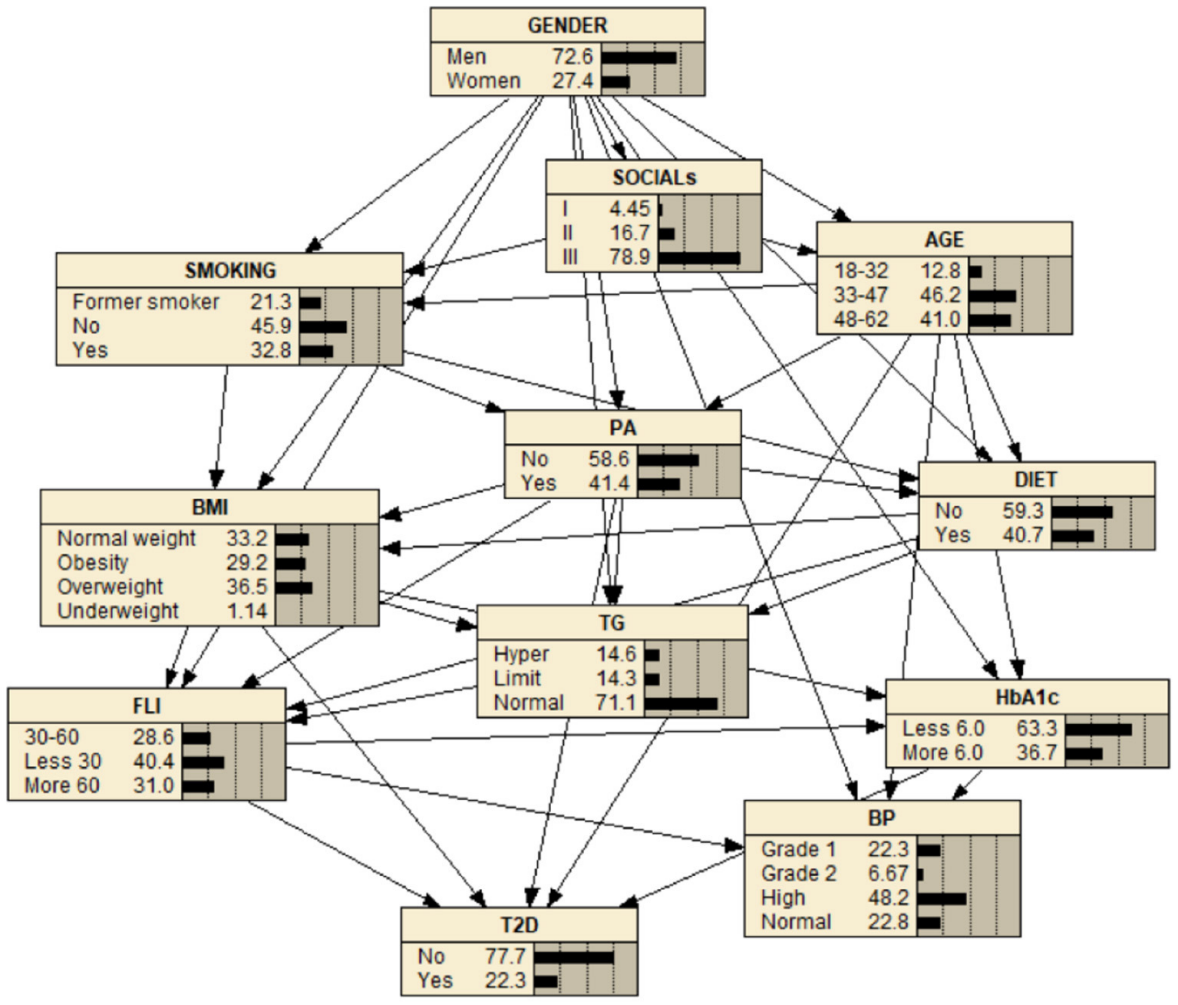
BN for the study of T2D. The BN shows high (48.2%) blood pressure (BP), normal (71.1%) triglycerides (TG), less 30 level (40.4%) FLI, overweight (36.5%) (BMI), less than 6.0 level HbA1c (63.3%), and practice physical activity (PA) (40.4%) and no practice physical activity (PA) (58.6%). It also shows a level of T2D equal to 22.3% in the yes state.

Once the BN is built (see Figures 2, 3), it is used to make inferences, i.e., probabilities are updated when new information is introduced (21). In order to make inferences, different reasoning patterns can be adopted (12, 14): *causal reasoning* (from top to bottom), *evidential reasoning* (from bottom to up), and *intercausal reasoning* (very close to human reasoning, it happens when different causes of the same effect can interact). The concept of the *Markov blanket* of a node [composed of its parents, its children, and the children's other parents (spouses)] is frequently used in order to reduce the features (variables) that may influence one another.

###  Analysis with the Markov blanket

In order to maximize the *T2D* variable in the *Yes* state, we considered the *Markov blanket* of *T2D* which is composed of *age, BMI, FLI, HbA1c*, and *PA* features, and we introduced evidence, selecting for each feature from the Markov blanket, the state which maximizes the most *T2D* in the *Yes* state (as *T2D* is on the bottom, a causal reasoning pattern is performed).

**Figure 4:**
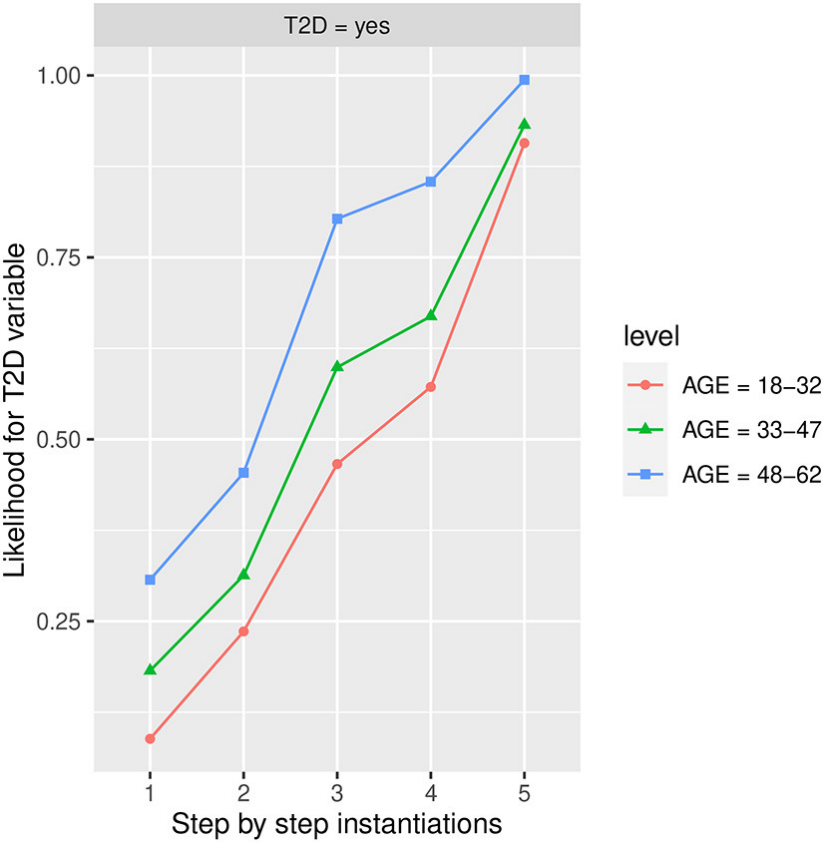
The different steps: step 1 = *Age*, step 2 = *PA* in the *No* state, step 3 = *BMI* in the *Obesity* state, step 4 = *FLI* in state *More than 60*, and step 5 = *HbA1c* in state *More than 6.0*, to evaluate *T2D* feature. The different steps are represented in the horizontal axis, while the estimated probability for the *T2D* variable at the value *Yes* is shown in the vertical axis.

**Figure 5:**
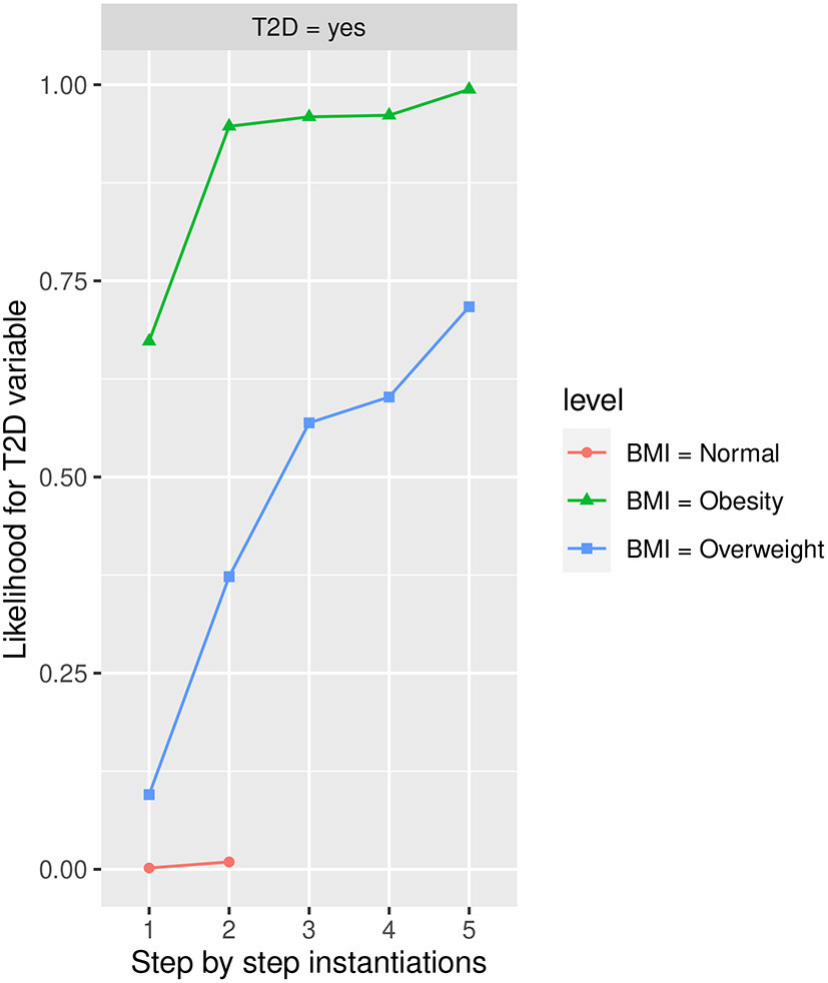
The different steps: step 1 = *BMI*, step 2 = *HbA1c* in state *More than 6*, step 3 = *FLI* in state *More than 60*, step 4 = *PA* in state *No*, and step 5 = *Age* in state *48-62* to evaluate *T2D* feature. The different steps are represented in the horizontal axis, while the estimated probability for the *T2D* variable at the value *Yes* is shown in the vertical axis.

**Figure 6:**
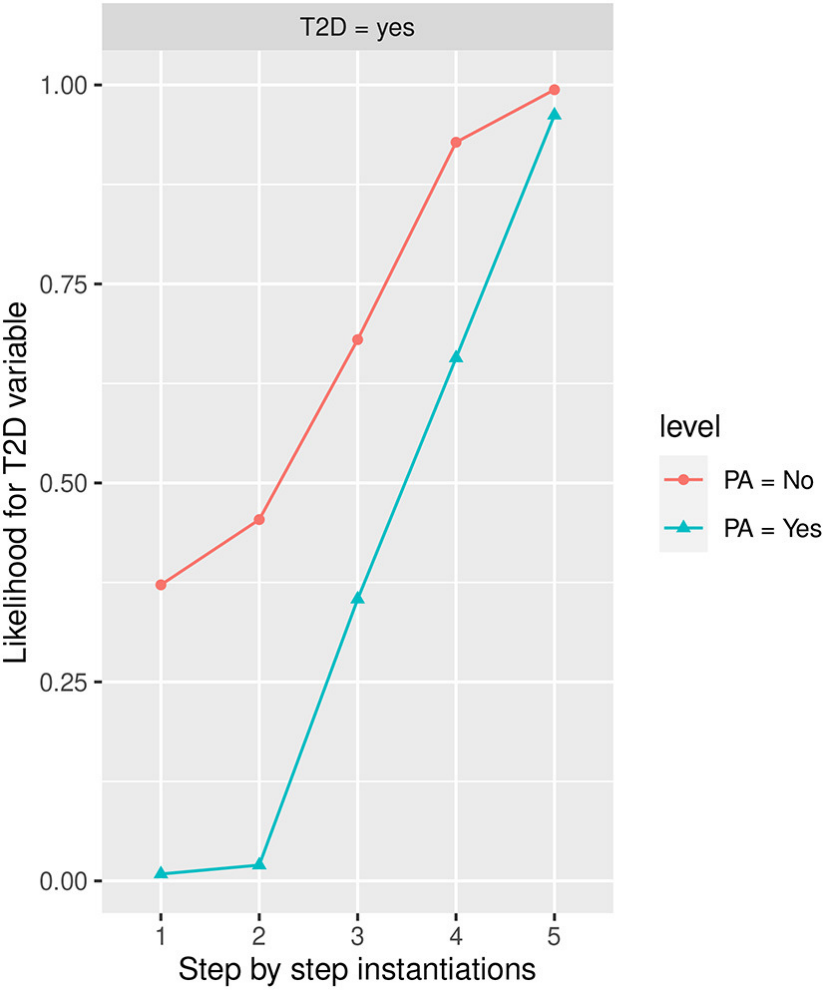
The different steps: step 1 = *PA*, step 2 = *AGE* in state *48-62*, step 3 = *FLI* in state *More than 60*, step 4 = *HbA1c* in state *More than 6.0*, and step 5 = *BMI* in state *Obesity* to evaluate *T2D* feature. The different steps are represented in the horizontal axis, while the estimated probability for the *T2D* variable at the value *Yes* is shown in the vertical axis.

**Figure 7:**
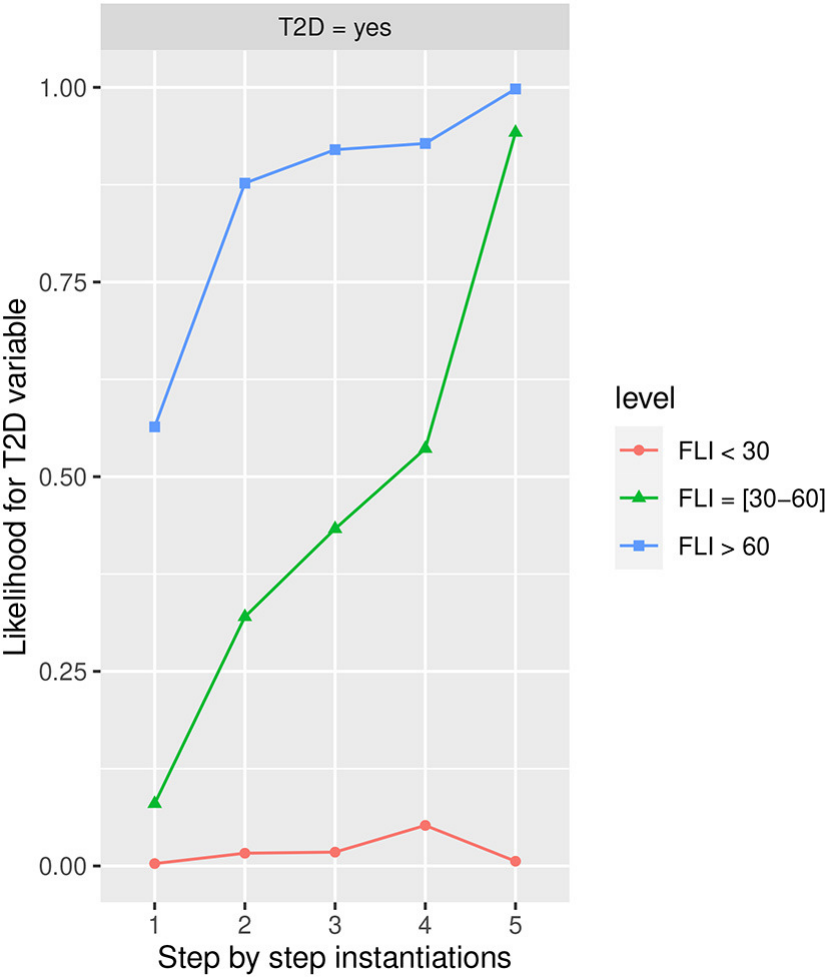
The different steps: step 1 = *FLI*, step 2 = *HbA1c* in state *More than 6.0*, step 3 = *AGE* in state *48-62*, step 4 = *PA* in the *No* state, and step 5 = *BMI* in the *Obesity* state, to evaluate *T2D* feature. The different steps are represented in the horizontal axis, while the estimated probability for the *T2D* variable at the value *Yes* is shown in the vertical axis.

**Figure 8:**
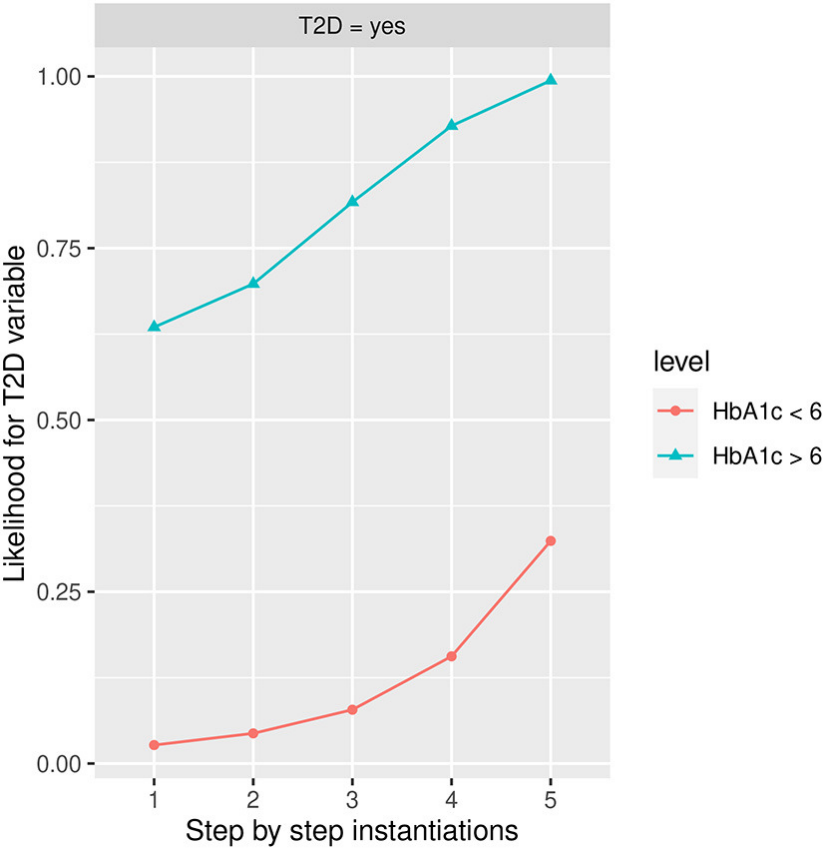
The different steps: step 1 = *HbA1c*, step 2 = *AGE* in state *48–62*, step 3 = *PA* in state *No*, step 4 = *FLI* in state *More than 60*, and step 5 = *BMI* in the *Obesity* state to evaluate *T2D* feature. The different steps are represented in the horizontal axis, while the estimated probability for *T2D* variable at the value *Yes* is shown in the vertical axis.

In Table 3, the features from the Markov blanket of *T2D* are ranked, showing the variable that increases the most *T2D* in the *Yes* state, being *BMI* in state *Obesity* which increases the most *T2D* in the *Yes* state going from an initial conditional probability value of 0.2230 to a 0.6730 value, followed by *HbA1c* in *more than 6.0* value increasing the conditional probability to 0.6350, and by *FLI* in *more than 60* value increasing the conditional probability to 0.5640, which is also shown in Figures 4–8 at step 1 in each of them.

In the following, a study of the likelihood variability for the *T2D* variable is considered taking into account the different states for each of the features (variables) that belong to the Markov blanket.

#### Influence of age on T2D

Figure 4 shows the likelihood variability for *T2D* in the different age groups, with 48–62 years being the age group that increases the most *T2D* in the *yes* state. Furthermore, as we observe in the groups of 18–32 and 33–47 years, it is the *HbA1c* in *more than 6* state which increases the most *T2D* in the *yes* state followed by *BMI* in the *Obesity* state. In group 48–62 is the feature *BMI* in *Obesity* state the one which increases the most *T2D* in *yes* state followed by *HbA1c* in *more than 6* state and *Physical Activity (PA)* in *No* state. In the age group 33–47, there is also a high influence of *BMI* in the *Obesity* state, it is observed that as the age decreases the influence of *BMI* in the *Obesity* state on *T2D* in the *yes* state also decreases. As we can observe, the group of 48–62 years is the one that increases the most *T2D* in the *yes* state, and therefore the one with the highest risk of developing *T2D*. Once *PA, BMI*, and *FLI* have been instantiated to *No, Obesity*, and *more than 60* states, respectively, the group of 18–32 goes from a conditional probability of *T2D* in the *Yes* state of 0.572 (57.2% expressed in percentage) to 0.907 (90.7% expressed in percentage) when the *HbA1c* is instantiated to *more than 6* state. In the case of the 33–47 group, this likelihood variates from 0.669 (66.9% expressed in percentage) to 0.932 (93.2% expressed in percentage).

#### Influence of BMI in T2D

In Figure 5, the likelihood variability for *T2D* in the different labels of BMI is shown, with *Obesity* being the one that increases the most *T2D* in the *yes* state, follow by *Overweight*. On other hand, no influence is shown in the groups for *BMI* in *Normal* and *Underweight* states. The highest influence in the groups of BMI for *Obesity* and *Overweight* states is obtained again when *HbA1c* is instantiated to *more than 6* state. The *Obesity* group is less influenced by the remaining instantiations while the *Overweight* group has still a high influence when *FLI* to *more than 60* state is instantiated, being also influenced (to a lesser extent) by *PA* in the *No* state and the *48–62* group of *age*. With both groups *BMI* in the *Obesity* state and in the *Overweight* state groups of risk, achieving a likelihood of having *T2D* in the *yes* state at the end of the instantiations was 0.994 (99.40% expressed in percentage) and 0.717 (71.70% expressed in percentage), respectively.

#### Influence of PA in T2D

Figure 6 shows the likelihood variability for *T2D* in the different labels of PA, the *No* state being the one that increases the most *T2D* in the *Yes* state. For those that practice PA, the highest influence in *T2D* (increasing its likelihood in yes state) is given by the *HbA1c* in *more than 6* state, the *BMI* in the *Obesity* state, and *FLI* in *more than 60* state, while for those that do not practice PA, the highest influence on T2D includes the *FLI* in *more than 60* state and the *HbA1c* in state *more than 6*.

In the worst-case scenario, i.e., when (*BMI*) is instantiated to the *Obesity* state, *HbA1c* is instantiated to *more than 6* state, *FLI* is instantiated to *more than 60*, and *age* is instantiated to *48-62*, the estimated conditional probability for those who practice PA achieves a value of 0.962 (96.2% expressed in percentage), while for those that do no practice PA achieves a value of 0.994 (99.4% expressed in percentage).

#### Influence of Fatty Liver Index in T2D

Figure 7 shows the likelihood variability for *T2D* in the different labels of *FLI*, with *more than 60* being the one that increases the most *T2D* in the *Yes* state, and therefore the one with highest risk of developing *T2D*. The *HbA1c* in *more than 6* state has the highest influence in all the groups determined by *FLI*, in the group *30–60, BMI* in the *Obesity* state also has a high influence.

Once the variables have been instantiated, the estimated conditional probability for *T2D* in the *yes* state is 0.0031 (0.31% expressed in percentage) in the group <*30*, 0.08 (8.00% expressed in percentage) in the group *30–60*, and 0.56 (56% expressed in percentage) in the group *more than 60* at step 1 in Figure 7, achieving at step 5 an estimated conditional probability of 0.0064 (0.64% expressed in percentage), 0.9420 (94.20% expressed in percentage), and 0.9940 (99.40% expressed in percentage), respectively.

#### Influence of HbA1c in T2D

Figure 8 shows the likelihood variability for *T2D* at the different labels of *HbA1c*, with *more than 6* being the one that increases the most *T2D* in the *Yes* state. The *BMI* in the *Obesity* state has a high influence on developing *T2D* in <*6* group followed by *FLI* in *more than 60* state and *PA* in the *No* state; while in the *more than 6* group, *PA* in the *No* state, *FLI* in *more than 60* state, and *BMI* in the *Obesity* state have a similar influence. Once the variables have been instantiated in step 1 the estimated conditional probability for *T2D* in the *yes* state is 0.0269 (2.69% expressed in percentage) in the group *HbA1c*< 6, and 0.6350 (63.50% expressed in percentage), in the group *HbA1c* >6, achieving at step 5 an estimated conditional probability of 0.3070 (30.70% expressed in percentage) and 0.994 (99.4% expressed in percentage), respectively.

###  Intercausal reasoning

The influence of some variables to reduce the risk of developing *T2D* is considered, taking into account that influence flows, in this sense, the Markov blanket cannot be completely considered, only some variables to allow the flow among variables.

#### Influence of diet and PA

**Figure 9:**
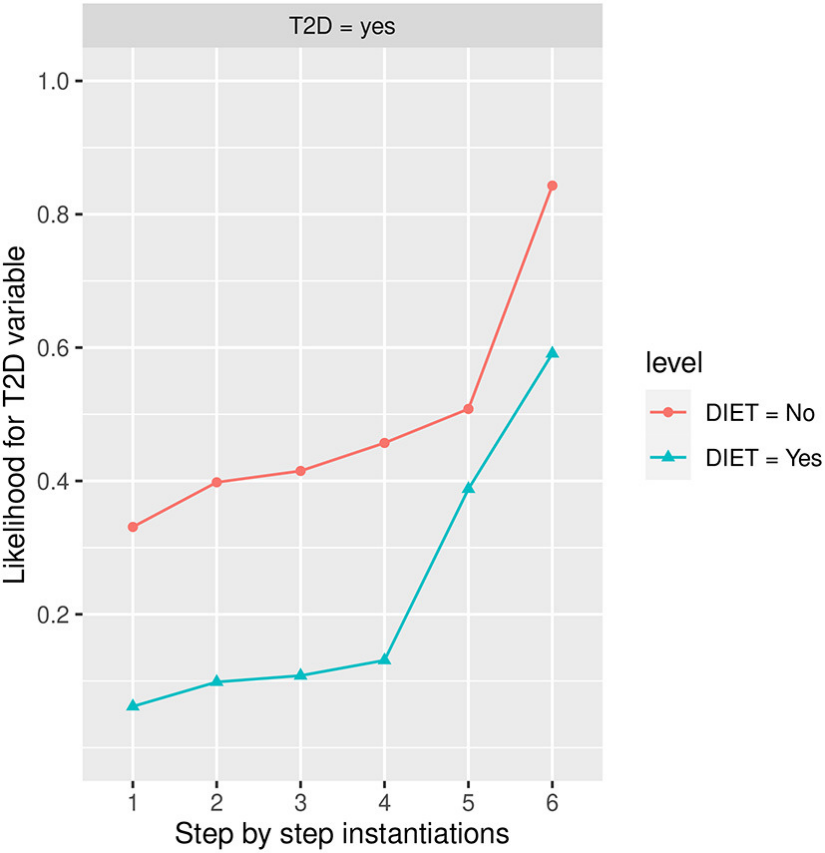
The different steps: step 1 = *DIET*, step 2 = *age* in state *48–62*, step 3 = *gender* in state *men*, step 4 = *SMOKING* in state *former smoker*, step 5 = *PA* in the *No* state, and step 6 = *BMI* in the *Obesity* state. The different steps are represented in the horizontal axis, while the estimated probability for *T2D* variable at the value *Yes* is shown in the vertical axis.

Figure 9 shows the influence of *Diet*, once *Diet* has been instantiated to the *No* state or the *Yes* state, the estimated likelihood of developing *T2D* is 0.3310 (33.10% expressed in percentage) and 0.0618 (6.18% expressed in percentage) respectively. The highest influence in the *No* diet group is given by *Body Mass Index* (*BMI*) in the *Obesity* state, while in the *Yes* diet group, it is given by *PA*, in the *No* state and *BMI* in the *Obesity* state. The risk of developing *T2D* is increased in both groups when *BMI* is instantiated to the Obesity state, reaching an estimated likelihood of developing *T2D* is 0.7130 (71.30% expressed in percentage) and 0.4660 (46.60% expressed in percentage) respectively, showing the strongest influence. Other factors such as *smoking* in the *former* state, *age* in *48-62* state, and *gender* in the *men* state increase the risk of developing *T2D*, reaching an estimated likelihood of developing *T2D* at step 5 of 0.8430 (84.30% expressed in percentage) and 0.5910 (59.10% expressed in percentage), respectively.

## Discussion

In this study, the feasibility of BNs in epidemiological studies is demonstrated, in particular when data from T2D risk factors are considered. Clinical questions based on unobserved evidence can be answered through specific BN models due to automatically updated probability distributions when new patient information is introduced.

The development and analysis of models to examine the relationships between different factors acting on T2D could be not only of theoretical interest but can serve as a generic tool for application oriented activities: explanation, prediction, monitoring, and prevention. BN models allow the theoretical analysis of the relationships between numerous variables in an appealing way, taking into account the probabilistic nature of the causal dependencies, in this sense, BNs constitute an adequate tool in the study of T2D. The ability of BN models of creating different scenarios based on hypothetical cases when new observations are considered to make BN models a powerful knowledge representation and an efficient reasoning tool under conditions of uncertainty. Furthermore, using the T2D model, a characterization of the whole set of variables could be given.

The main difference with respect to other T2D studies from prediabetes in the literature is that intercausal reasoning together with the concept of a Markov blanket were considered in order to optimize the T2D feature. The BN model is selected because they produce probability estimates rather than predictions. The process of learning the structure of a BN is a form of unsupervised learning, the learner does not distinguish the dependent variable from the independent ones, which is an advantage when compared with regression.

This longitudinal 5-year follow-up study evaluates risk factors for the progression from prediabetes to T2D among workers using a BN model and the *Markov blanket* concept. Our results showed that obesity and high levels of HbA1c are determinants for the progression to T2D. Furthermore, PA is an important protective factor even in the presence of other risk factors. The results of the present analysis are in accordance with previous evidence reporting that obesity is the main risk factor for T2D (8, 58, 59). Specifically, the risk for progression to T2D is very high (more than 50% risk) in prediabetic obese patients with and without high levels of HbA1c. However, high levels of HbA1c may help distinguish overweighted patients who will convert from those who will not. Similarly, when the instantiation begins with HbA1c, we also observe that obesity is a strong risk factor for conversion in subjects with high levels of HbA1c. But obesity and high levels of HbA1c are strongly associated; more than 60% of the patients who were obese also presented high levels of HbA1c. Notably, most of the normal weighted patients will not convert independently of the presence of other risk factors. High levels of FLI could also contribute to determining the risk of T2D, as it has also been previously described (60), especially in the case of overweight and obese patients. In addition, in overweight patients with high HbA1c levels, FLI (>60) strongly increases the risk of conversion to T2D. Interestingly, age is not a good predictor of developing T2D compared with other factors, and the main risk factors are important at all ages. In this way, our results highlighted that the main risk factors for conversion to T2D apply at different age groups with similar behavior at different steps and reaching a very high risk for progression to T2D (near 90% risk) independently of age. Although, some studies suggest that age could be a modest independent risk factor (61, 62), the practice of PA is an important lifestyle that could delay or avoid the progression to T2D in people with prediabetes, as previously described (8). We observed that people who practice and do not practice PA, obesity, HbA1c, and FLI are the factors that strongly increases T2D risk (according to the previously mentioned instantiation). Notably, even the presence of high levels of HbA1c is only an important risk factor for patients that do not practice PA. We used BN analysis to evaluate the influence of different variables in the progression to T2D from prediabetes. Compared with other types of analysis, such as logistic regression that also use the outcome as a binary, BN analysis evaluates the risk of different conditions (FLI, HbA1c, PA, and Diet), adding the presence of different variables instead of adjusting for the effect of other factors as logistic regression (63) that evaluate the adjusted effect of each condition. BN modeling is a more practical approach for clinical purposes since it allows more clinical use because it provides probability estimates for different scenarios that clinicians and patients could easily interpret. BNs could serve as a tool for helping clinicians in the management of risk factors assessment and clinical decision-making (14, 64). Early intervention is essential for T2D prevention, and BNs may allow clinicians to identify patients at high risk of developing T2D. For example, early intervention should be done without additional tests in patients with obesity, at a very high risk of conversion. But in overweight patients, clinicians should request additional tests to determine the real risk. It would not be necessary for patients with healthy weight because they are at low risk of conversion to T2D.

This study presents some limitations that should be acknowledged. First, a possible misclassification bias was when subjects were categorized as having prediabetes based on a single blood sample. Second, diet and PA were not evaluated with a validated questionnaire. On the other side, the main strength of the study was the large sample size with a 5-year follow-up. Furthermore, the study population was representative of the Spanish workforce.

## Conclusion

Our results confirm that obesity and high levels of HbA1c are the main risk factors for the progression to T2D, while PA is an important lifestyle protective factor. The BN analysis is an advanced model for dynamic description and prediction of the development of T2D. Furthermore, the BNs tool could be a feasible strategy to help clinicians with T2D prevention and motivate patients to adopt a healthier lifestyle that reduces their T2D risk.

## Data availability statement

The original contributions presented in the study are included in the article/supplementary material, further inquiries can be directed to the corresponding author.

## Ethics statement

The studies involving human participants were reviewed and approved by Institutional Review Board of the Balearic Islands Health 77 Research Ethics Committee (CEI-IB Ref. No: 1887). The patients/participants provided their written informed consent to participate in this study.

## Author contributions

PF-P, MB-V, and AY: conceptualization and writing original draft preparation. PF-P and MB-V: methodology. PF-P and AY: formal analysis. AL-G: investigation. AL-G and AA: data curation, writing review, and editing. All authors have read and agreed to the published version of the manuscript.
